# Small, N-Terminal Tags Activate Parkin E3 Ubiquitin Ligase Activity by Disrupting Its Autoinhibited Conformation

**DOI:** 10.1371/journal.pone.0034748

**Published:** 2012-04-04

**Authors:** Lynn Burchell, Viduth K. Chaugule, Helen Walden

**Affiliations:** Protein Structure and Function laboratory, The London Research Institute of Cancer Research UK, Lincoln's Inn Fields Laboratories, London, United Kingdom; University of Sheffield - MRC Centre for Developmental and Biomedical Genetics, United Kingdom

## Abstract

Parkin is an E3 ubiquitin ligase, mutations in which cause Autosomal Recessive Parkinson's Disease. Many studies aimed at understanding Parkin function, regulation and dysfunction are performed using N-terminal epitope tags. We report here that the use of small tags such as FLAG, cMyc and HA, influence the physical stability and activity of Parkin in and out of cells, perturbing the autoinhibited native state of Parkin, resulting in an active-for-autoubiquitination species.

## Introduction

**Figure 1:**
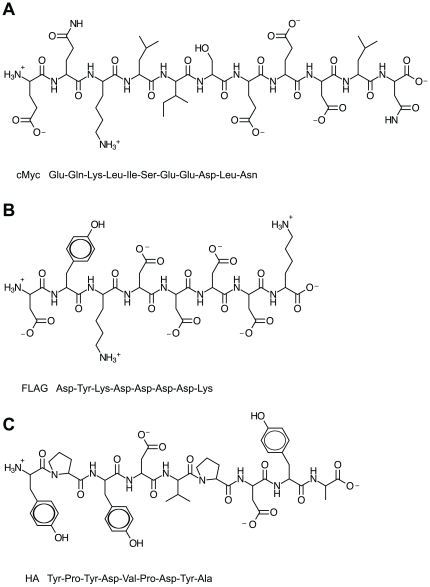
Chemical composition of small epitope tags. (**A**) The chemical composition of the cMyc tag, (**B**) the FLAG tag, and (**C**) the HA tag. The three-letter code amino acid sequence is shown beneath each linear representation.

Parkin is a RING E3 ubiquitin ligase [1], [2], [3] which, in conjunction with a ubiquitin-activating enzyme (E1) and a ubiquitin-conjugating enzyme (E2), catalyses the attachment of ubiquitin to itself and to multiple putative substrates [4], [5], [6]. Mutations occur throughout the gene sequence of Parkin, and include exon deletions, duplications and rearrangements, truncations, and point mutations. Mutations in Parkin account for ∼50% of Autosomal Recessive Juvenile Parkinson's Disease (ARJPD), making it the largest known hereditary factor in Parkinson's Disease [7], [8], [9], [10], [11]. Since the discovery of Parkin and its E3 ubiquitin ligase activity, Parkin has been the subject of intensive research efforts to understand its biology, substrates and consequences of pathogenic mutations. To date, Parkin is reported to have over 25 putative substrates, including itself, and has been shown to be regulated by an array of posttranslational modifications and interaction with deubiquitinases [12], [13], [14], [15], [16], [17], [18] and to mediate mitophagy [19], [20], [21], [22], [23]. Much of the research into Parkin, both in cells and *in vitro*, has been carried out using N-terminally tagged forms of Parkin. Depending on the study, the tag is to aid detection and visualisation, or the tag is a large protein fusion to aid in solubility and purification (for examples see [1], [2], [3], [13], [24], [25], [26], [27], [28], [29], [30], [31]). Fusion tags can vary greatly in size, from large tags such as Glutathione-S-Transferade (GST), Maltose Binding Protein (MBP) and Small Ubiquitin-like Modifier (SUMO), 26 kDa, 43 kDa and 13 kDa respectively, to small peptide tags including cMyc, FLAG and HA, each around 1 kDa (Figure 1). Recently, we demonstrated that wild type native Parkin exists in an autoinhibited state not competent for autoubiquitination [31]. Among other findings, we observed that MBP-, GST-, or SUMO-tagged Parkin was constitutively active, suggesting a disruption of the autoinhibited state. This was perhaps unsurprising given the large size of the fusion tags. However, the majority of cell-based studies of Parkin utilise small peptide epitope tags and the effect of these tags on Parkin activity and stability is unknown. Therefore we set out to determine the influence commonly-used cMyc, FLAG, and HA peptide tags have on the autoinhibited nature and stability of Parkin. We report here that small epitope tags fused to the N-terminus of Parkin also disrupt Parkin autoinhibition and Parkin stability.

## Results

### N-terminal tags reduce Parkin stability

**Figure 2:**
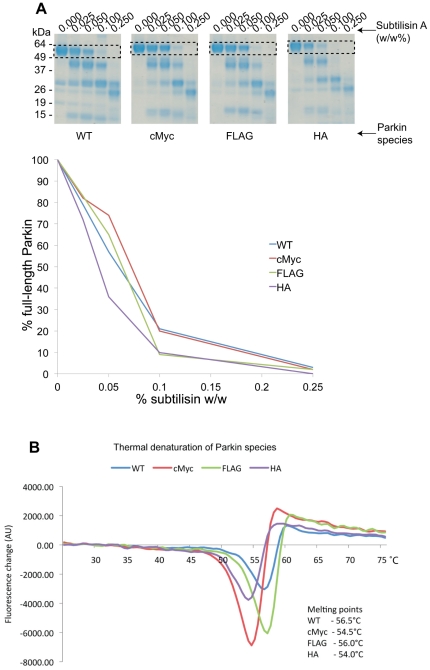
Epitope-tagged Parkin species are not in a native conformation. (**A**) Limited proteolytic digest of Parkin species in the presence of increasing concentrations of subtilisin (w/w). The boxed area indicates the degradation of each species. The graph below shows the quantification of the gels with each species normalised to the total amount of full-length species present in the absence of subtilisin. Each curve is coloured according to the key. (**B**) Difference scanning fluorimetry of each Parkin species, with each thermal denaturation curve coloured according to the key and melting points (Tm) of each protein indicated.

We expressed and purified to homogeneity cMyc-, FLAG-, and HA-tagged full length Parkin. In order to determine the effects of each peptide tag on the conformation and stability of Parkin, each protein was subjected to a limited proteolysis using subtilisin. cMyc- or FLAG-tagged Parkin are proteolysed in a similar pattern to wild type Parkin, suggesting that these tags do not impact the proteolytic susceptibility of Parkin (Figure 2A). In contrast, HA-tagged Parkin increases Parkin susceptibility to the protease, from the observation that HA-Parkin is digested at a lower concentration of protease in comparison to wild type Parkin. In addition to the limited proteolysis, we assayed the thermal stability of each species. A thermal denaturation experiment revealed the melting temperature of wild type, cMyc-, FLAG- and HA-tagged Parkin to be 56.5°C, 54.5°C, 56.0°C and 54.0°C respectively, revealing that cMyc- and HA-Parkin (but not FLAG-Parkin) have reduced thermal stability in comparison to wild type Parkin (Figure 2B). Taken together, these results indicate that the cMyc-, FLAG- and HA-tagged epitope tagged Parkin species are not in a fully native conformation as assessed by susceptibility to proteolysis and thermal denaturation.

### N-terminal tags release Parkin autoinhibition

**Figure 3:**
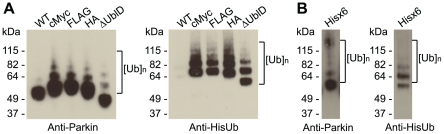
Epitope tags disrupt Parkin autoinhibition in vitro. (**A**) Western blot analysis of the autoubiquitination of wild type and cMyc-, FLAG, and HA-tagged Parkin. A truncation lacking the Ubl domain (ΔUblD) is the positive control. Ubiquitin conjugates are detected using anti-Parkin (left) and anti-His-Ub (right). (**B**) Western blot analysis of the autoubiquitination of Boston Biochem's His-Parkin, probed with anti Parkin (left) and anti-His-Ub (right). Ubiquitin conjugates are indicated.

Previous work from ourselves and others [26], [28], [31] has revealed that the N-terminal fusion of large proteins to Parkin results in a form of Parkin active for autoubiquitination. In order to test whether the same is true for small peptide tags we tested each species in an *in vitro* autoubiquitination assay. As expected, wild type native Parkin shows no autoubiquitination activity (Figure 3A). In contrast, cMyc-, FLAG- and HA-tagged Parkin are all heavily ubiquitinated, as seen by the formation of higher molecular weight species (Figure 3A). These data suggest that the presence of each of the N-terminal tags affects the autoinhibited state of the wild type protein. We also tested His-Parkin, commercially available from Boston Biochem. It is sold as a positive control for autoubiquitination and is purified in inclusion bodies and refolded (personal communication). In our hands it is active for autoubiquitination, as advertised (Figure 3B). This may be due to the disruption to the native state of Parkin caused by refolding, or due to the N-terminal His-tag.

### N-terminally tagged Parkin is active in cells

**Figure 4:**
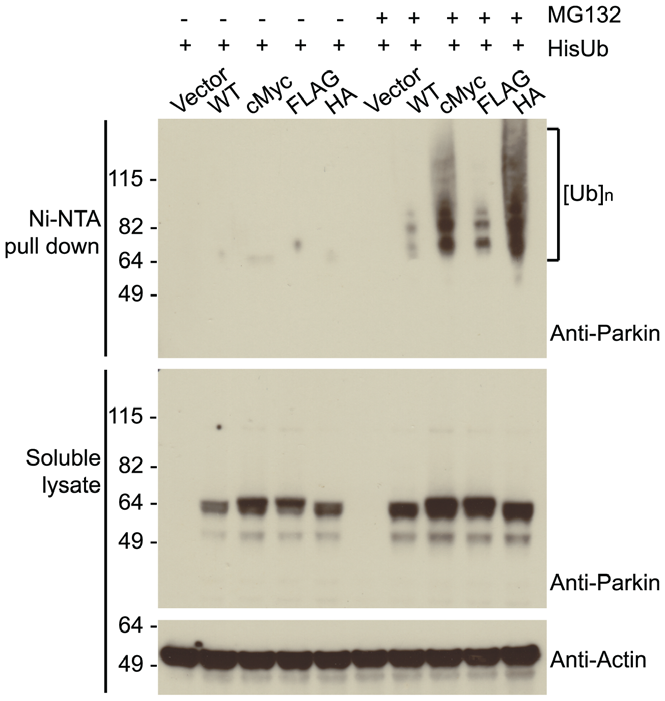
Epitope tags influence Parkin ubiquitination in cells. Western blot analysis of the ubiquitination of wild type and cMyc-, FLAG-, and HA-tagged Parkin in HEK293 cells. His-ubiquitin-conjugates were pulled out using nickel affinity and analysed using anti-Parkin (top panel). Soluble lysates were probed for levels of Parkin (middle panel) and actin levels are used as a loading control (bottom panel).

Given the effect on *in vitro* auto-ubiquitination of Parkin the N-terminal tags have, we hypothesised that the same would be true in an *in vivo* setting. To test this theory cMyc, FLAG and HA tags were cloned onto the N-terminus of wild type Parkin in a mammalian expression system. HEK293 cells were used to co-express these constructs along with His_6_-ubiquitin either in the presence of the proteasomal inhibitor MG132 or DMSO as a control. All ubiquitinated species were pulled out using nickel affinity chromatography and probed by western blotting using an anti-Parkin antibody to visualise any ubiquitination of Parkin (Figure 4, top panel). Analysis of the soluble lysates reveals how each of the different Parkin species are stabilised in the presence of MG132 (Figure 4, middle panel). Addition of proteasomal inhibitor also leads to the build up of ubiquitinated Parkin as seen by the high molecular weight laddering (Figure 4, top panel). Although wild type Parkin exhibits a small amount of ubiquitination, addition of the tags to the protein has a significant impact on the levels of ubiquitination seen; in particular, cMyc- and HA-tagged Parkin display high levels of ubiquitination relative to wild type untagged Parkin.

## Discussion

Many cell-based studies of Parkin function depend upon reliable detection by antibodies. One of the most established techniques for achieving high-affinity detection is to tag the protein of interest with an epitope recognised with high specificity by an antibody. As well as being useful tools in understanding biological processes, epitope tags are also physical and chemical entities. Our analysis clearly shows that N-terminal epitope tagging of Parkin, a commonly-used technique in the Parkin field, leads to physical changes in the stability and activity of Parkin, that are also observed in a cellular environment. Indeed, even modest changes in protein stability can translate to a more substantial impact on Parkin activity. Our work highlights the caveats involved in working with epitope-tagged Parkin, namely that it is not wild type. Previous work has specifically highlighted the ability of Parkin to use fusion tags as pseudosubstrates, for example MBP fused to Parkin has been shown to be ubiquitinated [28], [31]. This phenomenon is not unique to Parkin, or indeed E3 ligases, as a mass spectrometric analysis approach has also shown how fusion of a specific E2 to a GST-tag leads to ubiquitination of the GST tag on multiple lysine residues [32]. It is conceivable that there are more examples of tags being ubiquitinated that do not result in publication.

In our previous study we identified an interaction between the N-terminal ubiquitin-like (Ubl) domain of Parkin and a motif in the C-terminus [31]. It is feasible that the addition of a tag to the Ubl domain disrupts this intramolecular interaction, thus resulting in the release of the autoinhibition. Many studies that employ endogenous Parkin do not report the robust autoubiquitination of Parkin, in contrast to that seen in tagged-Parkin studies [15], [33], [34], [35], [36], [37]. In addition, there have been reports that Parkin can be alternatively transcribed after, or processed by cleavage of, the Ubl domain [38], [39]. When N-terminal epitope tags are employed, these species remain undetected.

Taking our results into consideration, efforts should be made to ensure the presence of a tag does not interfere with the native behavioural properties of the protein of interest. Results obtained using tagged proteins should be carefully controlled and interpreted with caution, taking into consideration changes to the protein's natural state upon addition of a fusion tag.

## Materials and Methods

### Expression and purification of proteins

cMyc, FLAG and HA were cloned directly onto the N-terminus of full length wild type Parkin in the pET SUMO vector using the Phusion® mutagenesis kit (Finnzymes). Tagged proteins were expressed in the same way as wild type Parkin, essentially as described in [31]. Briefly, Luria Broth growth medium was supplemented with 500 µM zinc chloride and cultures were grown at 37°C until OD_600_ 0.4. After reducing the temperature to 16°C, cells were grown to OD_600_ 0.8 before inducing with 25 µM Isopropyl β-D-1-thiogalactopyranoside (IPTG). Cells were harvested, sonicated and clarified before affinity purification using Ni-NTA resin (QIAGEN) and further purification on a gel filtration column following overnight cleavage with the SUMO protease, Ulp1. Proteins were flash frozen and stored in 50 mM Tris pH 8, 200 mM NaCl, 250 µM TCEP and 10% (v/v) glycerol.

### Proteolysis and thermal stability assays

Proteolytic susceptibility of each Parkin species was tested by adding 0.025, 0.050, 0.100 and 0.250 (w/w) % of Subtilisin A to 10 µg of Parkin in a total reaction volume of 20 µl. Reactions were incubated at 4°C for 3 hours before stopping with 20 µl of SDS-loading buffer. Gels were stained with SimplyBlue (Invitrogen).

Thermal stability assays were performed on an iQ5 iCycler (BioRad) in a 96 well plate format using a temperature gradient from 4°C to 100°C with 0.5°C increments. Each 100 µl reaction contained 5 µg of Parkin buffered with 50 mM Tris pH 7.5, 200 mM NaCl and 250 µM tris-2-carboxyethyl phosphine (TCEP). SYPRO Orange (Invitrogen) fluorescent dye was added to each reaction to a 1/200 dilution. Results are plotted in arbitrary units of the differential of the fluorescence divided by the differential of the temperature versus the temperature. The inflection point of the curve relates to the melting temperature of the protein.

### In vitro auto-ubiquitination assay

Autoubiquitination assays were carried out as described in [31]. Briefly, 15 nM UBE1, 1.1 µM UBE2L3, 5 µM His_6_-ubiquitin and 4 mM ATP in a reaction buffer consisting of 50 mM Tris pH 7.5, 2 mM DTT, 5 mM MgCl_2_ and 5% glycerol were mixed with 0.77 µM of each full length Parkin species before incubating at 37°C for 60 min. The 25 µl final reaction volume was stopped with 12.5 µl SDS-loading buffer. Samples were subjected to western blotting as follows: 1.3 µl loaded for visualisation with anti-Parkin (1/5000, 1A1, IBL) and 6 µl loaded for visualisation with anti-6xHis (1/2000, GE Healthcare).

### In vivo ubiquitination assay

Full length WT Parkin, cMyc-Parkin, FLAG-Parkin and HA-Parkin were cloned into the Gateway® destination vector pcDNA_DEST40 (Invitrogen) following the Gateway® technology manual. The His_6_-Ubiquitin mammalian construct was a kind gift from Dr Axel Behrens (London Research Institute). See [31] for detailed protocol. Briefly, HEK293 cells were transfected with 4 µg His_6_-Ubiquitin and 2 µg of either empty vector or a Parkin containing vector using Effectene reagents (QIAGEN) according to the manufacturers protocol. During the 48 hr incubation, cells were treated with either 10 µM DMSO or 10 µM MG132 (Calbiochem) for 12 hr. Clarified soluble lysates were collected and protein levels quantified. 100 µg of each sample was loaded onto a gel for western blotting analysis using anti-Parkin (1/5000, 1A1, IBL) and anti-Actin (1/400, abcam) as a loading control. His_6_-ubiquitinated species were affinity purified from soluble lysate containing a total of 7 mg of protein using Ni-NTA magnetic beads (QIAGEN). Samples were analysed by western blot using anti-6xHis (1/2000, GE Healthcare).

## References

[1] Zhang Y, Gao J, Chung KK, Huang H, Dawson VL (2000). Parkin functions as an E2-dependent ubiquitin- protein ligase and promotes the degradation of the synaptic vesicle-associated protein, CDCrel-1.. Proc Natl Acad Sci U S A.

[2] Shimura H, Hattori N, Kubo S, Mizuno Y, Asakawa S (2000). Familial Parkinson disease gene product, parkin, is a ubiquitin-protein ligase.. Nat Genet.

[3] Imai Y, Soda M, Takahashi R (2000). Parkin suppresses unfolded protein stress-induced cell death through its E3 ubiquitin-protein ligase activity.. J Biol Chem.

[4] Dawson TM, Dawson VL (2010). The role of parkin in familial and sporadic Parkinson's disease.. Mov Disord.

[5] Corti O, Brice A (2007). Of Parkin and Parkinson's: light and dark sides of a multifaceted E3 ubiquitin-protein ligase.. Drug Discov Today Dis Mech.

[6] Rankin CA, Roy A, Zhang Y, Richter M (2011). Parkin, A Top Level Manager in the Cell's Sanitation Department.. Open Biochem J.

[7] Martin I, Dawson VL, Dawson TM (2011). Recent advances in the genetics of Parkinson's disease.. Annu Rev Genomics Hum Genet.

[8] Hardy J (2010). Genetic analysis of pathways to Parkinson disease.. Neuron.

[9] Bonifati V (2012). Autosomal recessive parkinsonism.. Parkinsonism Relat Disord.

[10] Kitada T, Asakawa S, Hattori N, Matsumine H, Yamamura Y (1998). Mutations in the parkin gene cause autosomal recessive juvenile parkinsonism.. Nature.

[11] Lucking CB, Abbas N, Durr A, Bonifati V, Bonnet AM (1998). Homozygous deletions in parkin gene in European and North African families with autosomal recessive juvenile parkinsonism. The European Consortium on Genetic Susceptibility in Parkinson's Disease and the French Parkinson's Disease Genetics Study Group.. Lancet.

[12] Rubio de la Torre E, Gomez-Suaga P, Martinez-Salvador M, Hilfiker S (2011). Posttranslational modifications as versatile regulators of parkin function.. Curr Med Chem.

[13] Ko HS, Lee Y, Shin JH, Karuppagounder SS, Gadad BS (2010). Phosphorylation by the c-Abl protein tyrosine kinase inhibits parkin's ubiquitination and protective function.. Proc Natl Acad Sci U S A.

[14] Sha D, Chin LS, Li L (2010). Phosphorylation of parkin by Parkinson disease-linked kinase PINK1 activates parkin E3 ligase function and NF-kappaB signaling.. Hum Mol Genet.

[15] LaVoie MJ, Ostaszewski BL, Weihofen A, Schlossmacher MG, Selkoe DJ (2005). Dopamine covalently modifies and functionally inactivates parkin.. Nat Med.

[16] Yao D, Gu Z, Nakamura T, Shi ZQ, Ma Y (2004). Nitrosative stress linked to sporadic Parkinson's disease: S-nitrosylation of parkin regulates its E3 ubiquitin ligase activity.. Proc Natl Acad Sci U S A.

[17] Durcan TM, Kontogiannea M, Bedard N, Wing SS, Fon EA (2012). Ataxin-3 Deubiquitination Is Coupled to Parkin Ubiquitination via E2 Ubiquitin-conjugating Enzyme.. J Biol Chem.

[18] Durcan TM, Kontogiannea M, Thorarinsdottir T, Fallon L, Williams AJ (2011). The Machado-Joseph disease-associated mutant form of ataxin-3 regulates parkin ubiquitination and stability.. Hum Mol Genet.

[19] Gegg ME, Cooper JM, Chau KY, Rojo M, Schapira AH (2010). Mitofusin 1 and mitofusin 2 are ubiquitinated in a PINK1/parkin-dependent manner upon induction of mitophagy.. Hum Mol Genet.

[20] Geisler S, Holmstrom KM, Skujat D, Fiesel FC, Rothfuss OC (2010). PINK1/Parkin-mediated mitophagy is dependent on VDAC1 and p62/SQSTM1.. Nat Cell Biol.

[21] Vives-Bauza C, Zhou C, Huang Y, Cui M, de Vries RL (2010). PINK1-dependent recruitment of Parkin to mitochondria in mitophagy.. Proc Natl Acad Sci U S A.

[22] Youle RJ, Narendra DP (2011). Mechanisms of mitophagy.. Nat Rev Mol Cell Biol.

[23] Imai Y, Lu B (2011). Mitochondrial dynamics and mitophagy in Parkinson's disease: disordered cellular power plant becomes a big deal in a major movement disorder.. Curr Opin Neurobiol.

[24] Shin JH, Ko HS, Kang H, Lee Y, Lee YI (2011). PARIS (ZNF746) repression of PGC-1alpha contributes to neurodegeneration in Parkinson's disease.. Cell.

[25] Ren Y, Jiang H, Ma D, Nakaso K, Feng J (2011). Parkin degrades estrogen-related receptors to limit the expression of monoamine oxidases.. Hum Mol Genet.

[26] Matsuda N, Sato S, Shiba K, Okatsu K, Saisho K (2010). PINK1 stabilized by mitochondrial depolarization recruits Parkin to damaged mitochondria and activates latent Parkin for mitophagy.. J Cell Biol.

[27] Finney N, Walther F, Mantel PY, Stauffer D, Rovelli G (2003). The cellular protein level of parkin is regulated by its ubiquitin-like domain.. J Biol Chem.

[28] Matsuda N, Kitami T, Suzuki T, Mizuno Y, Hattori N (2006). Diverse effects of pathogenic mutations of Parkin that catalyze multiple monoubiquitylation in vitro.. J Biol Chem.

[29] Chew KC, Matsuda N, Saisho K, Lim GG, Chai C (2011). Parkin mediates apparent E2-independent monoubiquitination in vitro and contains an intrinsic activity that catalyzes polyubiquitination.. PLoS One.

[30] Wenzel DM, Lissounov A, Brzovic PS, Klevit RE (2011). UBCH7 reactivity profile reveals parkin and HHARI to be RING/HECT hybrids.. Nature.

[31] Chaugule VK, Burchell L, Barber KR, Sidhu A, Leslie SJ (2011). Autoregulation of Parkin activity through its ubiquitin-like domain.. Embo J.

[32] Cooper HJ, Heath JK, Jaffray E, Hay RT, Lam TT (2004). Identification of sites of ubiquitination in proteins: a fourier transform ion cyclotron resonance mass spectrometry approach.. Anal Chem.

[33] West AB, Gonzalez-de-Chavez F, Wilkes K, O'Farrell C, Farrer MJ (2003). Parkin is not regulated by the unfolded protein response in human neuroblastoma cells.. Neurosci Lett.

[34] Biasini E, Fioriti L, Ceglia I, Invernizzi R, Bertoli A (2004). Proteasome inhibition and aggregation in Parkinson's disease: a comparative study in untransfected and transfected cells.. J Neurochem.

[35] Joch M, Ase AR, Chen CX, MacDonald PA, Kontogiannea M (2007). Parkin-mediated monoubiquitination of the PDZ protein PICK1 regulates the activity of acid-sensing ion channels.. Mol Biol Cell.

[36] Van Humbeeck C, Waelkens E, Corti O, Brice A, Vandenberghe W (2008). Parkin occurs in a stable, non-covalent, approximately 110-kDa complex in brain.. Eur J Neurosci.

[37] Rakovic A, Grunewald A, Seibler P, Ramirez A, Kock N (2010). Effect of endogenous mutant and wild-type PINK1 on Parkin in fibroblasts from Parkinson disease patients.. Hum Mol Genet.

[38] Henn IH, Gostner JM, Lackner P, Tatzelt J, Winklhofer KF (2005). Pathogenic mutations inactivate parkin by distinct mechanisms.. J Neurochem.

[39] Schlossmacher MG, Frosch MP, Gai WP, Medina M, Sharma N (2002). Parkin localizes to the Lewy bodies of Parkinson disease and dementia with Lewy bodies.. Am J Pathol.

